# Considerations for Treatment in Clinical Care of Spinal Muscular Atrophy Patients

**DOI:** 10.3390/children11040495

**Published:** 2024-04-20

**Authors:** Stephanie Voight, Kapil Arya

**Affiliations:** 1 Department of Neuroscience, College of Natural Sciences, The University of Texas at Austin, Austin, TX 78712, USA; svoight@utexas.edu; 2 Division of Pediatric Neurology, Department of Pediatrics, University of Arkansas for Medical Sciences, Little Rock, AR 72202, USA

**Keywords:** spinal muscular atrophy, neurodegenerative, gene copies, nusinersen, onasemnogene abeparvovec, risdiplam

## Abstract

Spinal Muscular Atrophy is a neurodegenerative disease which can lead to muscle weakness, paralysis, and in some cases death. There are many factors that contribute to the severity of symptoms and those factors can be used to determine the best course of treatment for the patients. We looked through published literature to create a set of considerations for treatment in patients with Spinal Muscular Atrophy including age, type, SMN2 copies, and any familial considerations. This can serve as a guide for what to consider in the treatment of SMA patients clinically.

## 1. Introduction

Spinal Muscular Atrophy (SMA) is a neuromuscular condition that can lead to progressive weakness and an eventual loss of ambulation. It is also the leading genetic cause of death in infants. The disease is a result of insufficient amounts of the survival motor neuron protein (SMN) due to a mutation or deletion in the SMN1 gene. Humans have a variable number of SMN2 genes that code for a truncated version of the SMN protein that can help to reduce the severity of the SMA symptoms.

There have been various drugs approved by the Food and Drug Administration for the treatment of SMA. However, the use of these drugs depends on factors such as age, number of copies of SMN2, SMA type, and the use of any previous treatments. This paper will delve into the various considerations for treatment in individuals and provide an overview of the available treatment options by age group.

## 2. Results & Interpretations

Careful considerations must be taken when deciding on treatment for patients with spinal muscular atrophy. The approved treatments depend on the age of the patient and their number of copies of the SMN2 gene or the type of SMA that they have been diagnosed with. The different ages have been categorized based on available treatment options and study data. 

### 2.1. 0–2 Months Old

Diagnosis of SMA in patients at this age is most commonly done by prenatal testing, newborn screening, or infants who are beginning to display early symptoms. Early diagnosis is key and starting treatment early has been shown to help these individuals reach their motor milestones [1]. At this age, it is too early to determine the “Type” of SMA a patient may have, but the number of copies of the SMN2 gene has been shown to correlate well [2] (p. 1). The number of copies of the SMN2 gene that an individual may have needs to be taken into consideration when deciding on initial treatment for these patients. 

There are three FDA approved treatments for SMA at this age. These treatments include Onasemnogene abeparvovec (Zolgensma), Evrysdi (Risdiplam) and Nusinersen (Spinraza). All these treatments have been shown to benefit the patients in various ways and will be further discussed in the next three subsections.

#### 2.1.1. Nusinersen

Nusinersen is an antisense oligonucleotide drug that alters the slicing process to provide for longer versions of the SMN protein coded by the SMN2 gene. 

In the phase 2 NURTURE study (Table 1), nusinersen was given to infants who were presymptomatic and had a genetically confirmed diagnosis of SMA with 2 or 3 copies of SMN2 [3] (p. 2). The first dose was given at age 6 weeks or less and at the interim analysis, the infants had all surpassed the expected age of symptom onset for both Type 1 and Type 2 SMA [3] (p. 3). Following this there were seven individuals that developed SMA symptoms but continued to grow and increase scores in other assessments [4] (pp. 2–3). Efficacy was assessed in the infants using several different tests and baseline scores for each participant were gathered. There were 15 participants enrolled that had two copies and 10 participants that had three copies of SMN2. At the time of interim data analysis (approximately 3 years in), no individuals required permanent ventilation. This result remained true to the 5 year mark of the study [4] (p. 3). At both the 3 and 5 year mark, only 4 individuals (all with 2 copies) needed respiratory intervention [4] (p. 3). At the 5 year cut off, 80% of participants with 2 copies of SMN2 reached the highest score on the Children’s Hospital of Philadelphia Infant Test of Neuromuscular Disorders (CHOP INTEND) (motor function assessment) score and 100% of participants with 3 copies reached a maximum [4] (p. 3). All children with 3 copies of SMN2 achieved the WHO motor milestones within the normal timeframe [4] (p. 3). For the participants with 2 copies, all achieved sitting without support and standing without assistance. Fourteen could crawl and walk with assistance and 13 could walk alone [4] (p. 3). Throughout the study 40% had an adverse event (AE), though the majority resolved, except for 2 participant’s symptoms. 48% of the participants had a serious adverse event (SAE) though none were considered related to the study drug and no individuals chose to forego treatment [4] (p. 8). 

One important consideration for the use of nusinersen is the possibility of adverse effects including any administrative side effects. Nusinersen is given intrathecally, most often by a lumbar puncture. Many side effects are considered to be a result of this administration and include symptoms such as back pain, headache, and vomiting [5] (p. 8). There have also been reports of serious infections following administration [5] (p. 8). There is also the possibility of an immune response, though there have been no reported AEs in association to any anti-drug antibody findings [5] (p. 9). 

Nusinersen has been shown to delay the onset of symptoms as well as help individuals with SMA reach their motor milestones and maintain neuromuscular function. 

#### 2.1.2. Onasemnogene Abeparvovec

Onasemnogene abeparvovec (Zolgensma) is a gene therapy that provides a functional copy of the SMN1 gene that is either mutated or deleted in the patient. 

In the phase 3 SPR1NT trial (Table 2), Zolgensma was given to presymptomatic infants who had deletions of SMN1 and 2 or 3 copies of SMN2. 14 individuals with two copies were enrolled and given the gene therapy [1] (p. 3). The treatment was administered at a median day of life of 21 days [1] (p. 4). All children were able to sit independently, 11 of which reached this milestone in a normal time frame. All of the children could stand independently and 50% achieved this in less than 540 days [1] (p. 4). Based on the WHO Multicenter growth reference sheet (WHO-MGRS), 71% of the children could walk alone, and 6 individuals could do it in the expected time frame. At the end of the study, no children required permanent ventilation and no child needed any respiratory support throughout the study [1] (p. 4). All of the participants with 2 copies achieved a CHOP INTEND score of at least 58 by the end of 18 months [1] (p. 5). There were minimal elevations in liver enzymes levels and platelet counts decreased in some individuals. There were also 3 individuals with sensory affects, however 2 of these resolved [1] (pp. 8–9). There were no associated cardiac dysfunction or events of thrombosis [1] (p. 6). 

For the same SPR1NT trial, 15 participants with three copies of SMN2 were enrolled. Each participant was given the treatment at a median age of 32 days [1] (p. 2). All 15 of the enrolled participants were able to stand alone, 14 of which did so in the expected normal time frame. 93% of the children were able to walk independently by 2 years old. At the end of the trial, no children required permanent ventilation and no child required any mechanical respiratory support at any point during the trial [1] (p. 3). The motor scale used for the individuals with 3 copies was the BSID which was then converted to a scaled score. At the two year visit, all children had reached the scaled score greater than or equal to 4 with the median at 9 (very close to the score of those individuals without SMA) [1] (p. 3). Each participant did have at least 1 EA though few were serious, and none were related to the treatment.

There are a few important aspects to consider when deciding on Zolgensma. The first is that it is a one-time administration intravenously. This can help to reduce the need to travel to and from the facility aside from any follow up. There is also the need to consider that there is the risk of immune response in gene therapies. However, SPR1NT has shown that if given early the safety of the administration is favorable [1]. This is because in newborns the immune system is fairly tolerant to non-self antigen [1] (p. 8).

There are a few additional considerations for patients in this age range. The first is that these are likely treatment naïve patients that have not been on any previous medications. It is best to evaluate case by case and discuss with the family which treatment option seems more fitting for their family. 

Another consideration is for the infants that have deletions or mutations of SMN1 with 4 copies of SMN2. There is no trial data for these individuals and so these patients should be considered on a case-by-case basis. There is some debate over whether the best practice is treating these patients proactively or whether treating these patients is creating unnecessary burdens [6] (p. 2). However, there is support for treating these patients following early diagnosis to prevent the development of any SMA symptoms. Discussions with the family can be used to find the best option.

#### 2.1.3. Risdiplam

Risdiplam is a small molecule and functions as an SMN2 splice modulator to include exon 7 in preRNA. RAINBOWFISH (Table 3) is an open-label, single-arm, multinational study phase 2 clinical trial of genetically diagnosed presymptomatic infants with SMA treated with risdiplam up to 6 weeks of age. 7 patients (four patients had two SMN2 copies, two patients had three SMN2 copies, and one patient had four SMN2 copies or more) were studied. Of six patients with two or three SMN2 copies 100% achieved sitting; 67% could stand, and 50% patients could walk independently as measured by the HINE-2 at Month 12. All participants had event-free survival at 12 months [7,8,9].

Following this, many centers have anecdotal reports of effective and safe use of risdiplam in neonates and infants up to 2 months old.

### 2.2. 2–6 Months Old

Diagnosis of these patients is most likely due to the development of symptoms. Historically, development of symptoms this early would lead to a “Type 1” diagnosis. There are still a few key considerations for treatments in this age range including SMN2 copy number and symptom severity. 

The three medications previously discussed, Nusinersen, Risdiplam and Onasemnogene abeparvovec, are approved in this age as well. 

#### 2.2.1. Nusinersen

The efficacy of Nusinersen was evaluated in the phase 3 ENDEAR trial. Participants in the trial were 6 months or younger at the onset of their symptoms and had 2 copies of SMN2 [10] (p. 2). Interim analysis was done after 80 infants had been enrolled for at least 6 months and at that time nusinersen showed favorable results for the drug. At the analysis cut off point 41% of the nusinersen group showed a motor- milestone improvement (increase in HINE-2) as compared to the 0% in the sham procedure group [10] (p. 10). The risk of death or permanent ventilation was also much lower in the nusinersen group compared to the sham group [10] (p. 5). The nusinersen group also showed a higher CHOP INTEND scores as compared to the control group [10] (p. 5). Of the SAEs that occurred, there were a lower number in the nusinersen group than the control group [10] (p. 7). These numbers included the events that led to discontinuation in the trial, all of which were fatal in outcome [10]. The infants were closely monitored after administration and no safety concerns were noted during this time [10] (p. 9).

As noted previously, many of the side effects of the medication have to do with the route of administration. Many of these are not noted in such a young population as the infants in the trial were nonverbal [10] (p. 9). The same concerns remain for the treatment as those in the 0–2-month age category. 

#### 2.2.2. Onasemnogene Abeparvovec

Two different clinical trials showed the efficacy of the gene therapy in infants throughout this age range. START (Table 4) is an ongoing phase 1 clinical trial testing the safety and the lasting efficacy of the therapy in treatment of individuals with Type 1 SMA and 2 copies of SMN2 [11] (p. 3). Of the 10 individuals in the therapeutic dose cohort, none required permanent ventilation and 2 of the three in the low dose did not require permanent ventilation [11] (p. 4). At enrollment, 6 of the 10 in the therapeutic dose did not require any assisted ventilation and this remained true at the interim analysis point. All 10 of the therapeutic dose group were able to stand assisted and 8 of them had done this prior to the interim analysis cut-off [11] (p. 4). Out of the 13 enrolled, 8 total patients had a serious adverse effect (1 of which was in the low-dose cohort). Most often, the SAEs reported had to do with the disease of SMA specifically [11] (p. 4). This study showed a favorable safety profile all the way to around 6 years after dosing and all SAEs were expected based on other Onasemnogene abeparvovec trials [11] (p. 6). 

The other clinical trial that evaluated the efficacy was the STR1VE phase 3 trial (Table 5). Patients must have been under 6 months old and have 1 or 2 copies of SMN2. It was found in this study that 59% of the participants were able to sit on their own by 18 months. It was also found that 20/22 patients did not need permanent ventilation [1]. Every patient experienced an SAE, though only 3 were thought to be or were related directly to the treatment [1]. The risk-benefit profile was in support of treatment of presymptomatic or symptomatic individuals likely to develop Type 1 with the gene therapy [1].

There are still the same concerns and considerations for these patients as well as the infants from 0–2 months when considering the use of Zolgensma. There is still the risk of immune response once given the gene therapy treatment. It is also important to consider the families capacity for follow up visits and how often they would like to travel to the hospital. 

#### 2.2.3. Risdiplam

In the FIREFISH pt. 2 study (Table 6), researchers evaluated the efficacy of the treatment in patients with Type 1. There were 41 individuals enrolled in the Type 1 study and at the 2-year mark, 18/38 on going participants were able to sit independently [8] (p. 4). The patients receiving medication did continue to improve on their motor assessments and reach motor milestones over time. However, none of these patients were able to stand or walk on their own [8] (p. 9). There were 68 SAEs reported for 28 infants. Seven infants experienced an adverse effect contributed to the treatment. No participants left the study due to an event and only one patient had to have dosing interrupted [8] (p. 9). 

An important distinction to make about risdiplam is that it is taken once daily at home orally. This can provide ease of accessibility and might help to keep patients more adherent to their schedule. This medication could also help to alleviate the burden of regular visits to the hospital if that is a hindrance within a family. 

Just as before, there is still the need to consider that these are likely patients who have not previously received treatment. Informing the family about each of the treatment options is important and helping them to understand the pros and cons of each is vital to ensuring the best decision for the patient is made. 

Since the medications have shown that treatment type and response can vary by type or copy number, it is important to consider the number of SMN2 copies the patient has when discussing treatments. It may also be important to consider the onset and severity of their symptoms to determine how rapidly progressing the disease is. 

### 2.3. 6–24 Months Old

Patients in this age range are receiving a diagnosis most likely due to symptom onset. At this age, onset of symptoms would indicate that the type of SMA is likely 2 or 3 though it could be a late onset of type 1. The type of SMA can once again help to indicate which treatment options might be best out of the three available for this age range. 

#### 2.3.1. Nusinersen

The ENDEAR trial (discussed for 2-6 months), has shown that patients with nusinersen have a better likelihood of not needing permanent ventilation and will be more likely to meet their motor milestones and not have regression in their motor function [10]. 

The concerns with this treatment remain the same as other age groups taking the medication. 

#### 2.3.2. Onasemnogene Abeparvovec

Both the START and STR1VE have shown that Zolgensma can help to avoid permanent ventilation in patients that otherwise likely would have needed it. The gene therapy can also help those same individuals meet motor milestones and improve their motor function [1,11]. 

The risks and benefits hold true throughout this age group. At this age, it becomes a slightly higher risk for gene therapies as the immune system continues to develop. However, this is still within the safety range of the drug. 

#### 2.3.3. Risdiplam

As shown in the FIREFISH study, the oral medication can also help to reach motor milestones and to improve the overall motor function [8]. There was no study data specific for just this age group, however, more information about the use of Risdiplam in various types of SMA could be helpful in determining the best treatment for patients. 

At this age, patients may be medication naïve, but some patients may also be looking to switch medications. Some may even look to combine treatments in some way. There has not been much research done for the combination of treatments, particularly any combining with Zolgensma. Just as before, it is important to consider the severity of symptoms when deciding on a treatment plan for these patients as well as any non-physiological concerns they may have about the different options. 

### 2.4. 2–15 Years Old

Patients in this age range will rarely be a new diagnosis and they are most likely Type 2 or Type 3. This age range is capped at 15 years old tin order to best discuss trial data from the CS2/CS12. At this age, gene therapy is no longer an available therapy, so the two available treatments are Nusinersen and Risdiplam. 

#### 2.4.1. Nusinersen

In the CHERISH phase 3 trial, efficacy of the drug in individuals with later onset symptoms was evaluated and compared to individuals not receiving the drug. Eligibility was determined by symptom onset after 6 months of age and then screening between the ages of 2 to 12 years old. The child had to be able to sit independently but have no history of ever being able to walk independently. The child also had to meet test scores for the HFMSE, never have been diagnosed with scoliosis, and must not have respiratory or nutrient intake insufficiency [12] (p. 3). 126 individuals enrolled and at the time of interim analysis (the trial ended here), 100 children had completed the trial (66 nusinersen and 33 control). Overall, the study showed that those in the nusinersen group had an increase in their motor function and they did not experience the average decline seen in the control patients [12] (p. 4). The motor milestones also showed no decline in the nusinersen group, though there was no difference in the groups for those that could stand or walk alone [12] (p. 5). Of the serious adverse events that occurred, the percentage was higher in the control group and some of the events might not be related to the treatment [12] (p. 5). 

In the CS2/CS12 phase 1 and 2 trial, the efficacy of the treatment was evaluated in 28 children. Only those who had completed the CS2 study were eligible for the CS12. Of the 34 who enrolled in CS2, 24 went on to CS12. These children were all between the ages of 2–15 years old [13] (p. 4). 39% had Type 2 and 61% had Type 3 SMA [13] (p. 6). The children with Type 2 showed improvement in their motor function while those with Type 3 had a modest change [13] (p. 6). One of the children in the type 2 category gained the ability to walk, and 2 in the Type 3 ability were able to regain their ability [13] (p. 8). All children in the trial had at least one adverse event, many of which related to the lumbar puncture. Of the serious events that occurred, none were determined to be related to the drug [13] (p. 8). 

These studies show that nusinersen can be beneficial in the older patients with SMA. The drug has been shown to help improve motor function, stabilize the progression of symptoms, and in some cases allowed individuals to reach new motor milestones. 

#### 2.4.2. Risdiplam

The safety and efficacy of Risdiplam in older individuals was evaluated in the SUNFISH phase 3 trial. Participants in the trial had to be between the age of 2 and 25 and must have type 2 or type 3 SMA. Patients had to be able to sit independently, not have the ability to ambulate, and had certain score criteria they must meet for motor function [14]. individuals received risdiplam and 59 patients were in the control group. 5% more individuals who received the drug had adverse effects though the series adverse effects were consistent across groups. The study showed that risdiplam provided for a significant improvement when compared to the control group. Younger individuals showed more improvement while older individuals showed a stabilization and no significant decreases [14]. 

At this age, it is important not only to consider how the drug might be able to improve motor function or help patients reach motor milestones but also consider how the treatment might stabilize any decline in motor functions. Overall, both medications in these age ranges have shown to help stabilize the progression of the disease in older patients and contribute to the advancements in the scores of younger individuals. 

This is also an age that patients may be looking to combine or switch between medications. It is important to discuss these options with the family and see current research on the benefits of combinations of treatment. 

### 2.5. 15 Years Old-Adulthood

The final age group to consider are the patients who are 15 years or older. There will very rarely be any new diagnoses at this age. Majority of the patients seeking treatment at this age will be Type 2 or Type 3, though there may also be Type 1 patients on permanent ventilation. When deciding on treatments for these individuals, similar considerations may apply including type, severity of symptoms, and any previous treatments the patient may have used. Data detailing the impact of treatments on adults is scarce though as the years progress, ideally more information will become available to allow for a clear decision-making process. 

#### 2.5.1. Nusinersen

An observational study was completed by Hagenacker to evaluate the impact that Nusinersen had in older children to adults living with SMA. The data was broken down into month analyses and looked at the change in the Hammersmith motor functional scale (HFMSE) [15] (p. 2). The paper also found that with an increase of 3 points or more to the scale, these improvements were stable for up to 14 months after [15] (p. 4). The greatest improvements were in those with less severe symptoms; however, improvements were seen all around [15] (p. 6). 

The same safety concerns remain for adults receiving Spinraza as those discussed in other sections of the paper. This most common adverse effect throughout all the ages is the lumbar puncture associated symptoms. 

#### 2.5.2. Risdiplam

The data in support of Risdiplam for this age group is the same as the data in support for age 2–15. The SUNFISH trial evaluated the medication on patients up to age 25, but it can be used for individuals at older ages [14]. 

The clinical concerns for risdiplam still apply within this age group as do the associated benefits including the ease of administration. 

Just as in the patients from 2 to 15, stabilization of symptoms is a very important factor to consider when deciding on clinical treatment. Prevention of any further decline can help these patients to maintain some independence and not have any additional care taking needs that can ultimately put strain on families. 

In this age range, it is important to consider the severity of symptoms as these may impact further treatment decisions. For example, if a patient is not able to sit on their own, they might also have a diagnosis of scoliosis or impacts on their respiratory status. Considering the patient’s ability for access is an important part of clinical care and can help to alleviate any extra stress on families when moving forward with treatment options. Patients who are sitters might still have scoliosis and had surgeries such as rods or vertebral fusions to help those conditions. Access for these patients is also a consideration that must occur to ensure the best clinical care. Finally, a patient might have the ability to walk. These patients most often have the strongest phenotype. The patient’s ability to walk might still be enhanced with treatment or with the decision to provide access to a walker. These are all important conversations that would need to be discussed with the patient and their family to determine the best course of action. 

## 3. Conclusions

Overall, the various treatments used for SMA have been shown to not only stabilize the symptoms but also to help improve motor milestones and motor function in many younger individuals. These treatment options each have their benefits and cons, so it becomes vital to weigh the options and consider all the information available. The type of SMA, number of SMN2 copies, respiratory status, and severity of symptoms are all important to consider. Further exploration and research is needed to have scientific validation of improvement in function with combined or sequential use of a combination of onasemnogene and risdiplam or onasemnogene and nusinersen, although some initial evidence does indicate that such an approach may be safe and clinically beneficial to patients [16,17,18,19].

## Figures and Tables

**Table 1:** Data from Nurture Trial [3,4]. Total number of participants was 25. All data is from the update after 5 years.

	2 Copies of SMN2 (n = 15)		3 Copies of SMN3 (n = 10)	
Permanent Ventilation	None		None	
Respiratory Intervention (percent)	27%		0%	
Reached maximum CHOP-INTEND score (percent)	80%		100%	
WHO Motor Milestones achieved at any point (percent)	Sitting without Support	100%	Sitting without Support	100%
	Standing with assistance	100%	Standing with assistance	100%
	Hands/knees crawling	93%	Hands/knees crawling	100%
	Walking with assistance	93%	Walking with assistance	90%
	Standing alone	87%	Standing alone	100%
	Walking Alone	87%	Walking Alone	100%

**Table 2:** Data from SPR1NT Trial [1]. Total number of participants was 29.

	2 Copies of SMN2 (n = 14)		3 Copies of SMN2 (n = 15)	
Permanent Ventilation (percent)	0%		0%	
Respiratory Intervention (percent)	0%		0%	
Highest CHOP-INTEND Score achieved by all participants	58		---	
WHO-MGRS achieved at any point (percent)	Sitting without Support	100%	Sitting without Support	100%
	Standing alone	---	Standing alone	100%
	Walking Alone	71%	Walking Alone	93%

**Table 3:** Data from RAINBOWFISH. Data collected at month 12.

		2 Copies of SMN2 (n = 4)	3 Copies of SMN2 (n = 2)	4 or More Copies of SMN2 (n = 1)
HINE-2	Sit without support	100%		---
	Stand alone	67%		
	Walk alone	50%		

**Table 4:** Data from START (n = 13).

	Therapeutic Dose (n = 10)	Low Dose (n = 3)
Permanent Ventilation Required? (percent)	0%	33%
Ventilation Assistance (percent)	40%	---
Able to Stand Assisted (percent)	100%	---

**Table 5:** Data from Strive (n = 22).

	1 & 2 Copies SMN2
Permanent Ventilation Required? (percent)	9%
Able to Sit Alone (percent)	59%

**Table 6:** Data from FIREFISH (n = 38 ongoing).

	SMA Type 1
Able to Sit Alone (percent)	47%

## Data Availability

No new data were created or analyzed in this study. Data sharing is not applicable to this article.
